# Illinois State Dental Society

**Published:** 1884-08

**Authors:** 


### ILLINOIS STATE DENTAL SOCIETY.

TWENTIETH ANNUAL MEETING.

REPORTED EXPRESSLY FOR THE INDEPENDENT PRACTITIONER.

(Continued from page 380.)

*Dr. G. V. Black*—Said that he was already on record on the question of capping pulps, for he had expressed himself at former meetings. His earlier experiments in this pulp matter were not very successful. He would suggest that we differentiate, for cases vary in pathological condition. Thus, Dr. Crouse says if the pulp is normally sensitive he decides to cap. I will only add that if I see an abnormal condition, I decide not to risk capping. I would hesitate more in a child's case than in that of an adult. The pulp is less and less important with age, and more important with a child. One can cap the pulp in the mouth of a child with much better results than with an adult. Something has been said about the coagulum. I had rather not have that coagulum. If the parts are healthy, the removal may be brought about by absorption. The removal of the blood clot there by absorption is not different from the removal of a clot in the tissues anywhere.

As to the manner of capping, my practice has been to avoid irritation ; hence I abandoned the use of chloride of zinc, and also the use of carbolic acid in large enough quantities to irritate. A greater per cent of success is attainable, I am persuaded, without forming a coagulum, by using about a thirty per cent solution of carbolic acid, then oxy-phosphate of zinc for the cap. I use a little piece of paper and drop on this the preparation of oxy-phosphate; then carry it into the tooth and lay it there. The warmth distributes it evenly. I think that makes the nicest cap. Then I use wintergreen and oil of cinnamon.

*Dr. Sitherwood*—Said that success in capping depended to a great extent upon good instruments, and mechanical skill and dexterity in using them. He used Listerine as an antiseptic, in preference to carbolic acid. A leading physician of his acquaintance had used this remedy very successfully in irritations of the mucous membrane, and thus he was led to use it and to commend it.

*Dr. Newkirk*—The condition of the blood naturally affects the tooth, as it does other parts. What is the condition of the pulp? That is the question, and it is the principal consideration to govern in the operation. Skill and judgment are requisite, but if the conditions are bad they will not avail, and the pulp will die in any case. Put a little oil on the probe; it will go easier. With regard to instruments, I fear we use sharp points too much. I would have a curved edge; perhaps I should say spoon-shaped. No pulp should be capped if hemorrhage exists, otherwise we have a phase of embolism. It is important to delay capping until hemorrhage ceases; then nature may be trusted to take care of the matter.

*Dr. L. C. Ingersoll*—Spoke of the objection urged against the use of arsenious acid, and said the after results must necessarily depend upon the after treatment. I apply dialysed iron to remove the effect of the arsenious acid, in fact to antidote this poison. We have, in my judgment, no antiseptic equal to arsenious acid as a preservative. I could instance a case where I found upon examination, after a long absence, that the pulp was preserved absolutely perfect.

*President Noyes*—I would ask if the dialysed iron entirely removes the effect of the arsenious acid and how?

*Dr. Ingersoll*—I cannot precisely explain the process, but I know that it does it. The dialysed iron is laid down in the books as an antidote for arsenious acid.

*Dr. Brophy*—Said a tooth properly and skillfully filled was much better of course than one carelessly treated. It was a source of much pleasure to him to confirm the experience of Dr. Black. He too, opposes the use of oxy-chloride of zinc, as not compatible with the parts. He has removed many caps where it has been used, and found dead pulps. As to the use of carbolic acid, he does not see the necessity for producing coagulum. Why should we destroy to make well? Thinks we ought to avoid the use of everything tending to inflammation and irritation. As Dr. Newkirk has well said, we might use a spoon-shaped instrument. His idea is good. And, as Dr. Sitherwood has said, Listerine is an excellent antiseptic. Temporary dressing of the exposed part, so that it can subsequently be capped, is, in his judgment, an effective and practical treatment.

Does not believe in the sometimes received and advocated opinion that the application of arsenious acid should be removed in twenty-four hours. Thinks forty-eight hours, or even two or three days will do better.

*Dr. Spalding*—Do you think a week any too long?

*Dr. Brophy*—None too long, sir, nor even two weeks.

*Dr. Spalding*—That's true.

*Dr. Brophy* (continuing)—I am inclined to commend the use of a heated instrument—heated to a white heat—to insert in the canal. Of course there is necessity to use it very speedily. The process is quick and painless. I favor, as a general practice, the saving of small portions of the pulp.

*Dr. Ingersoll*—The main question that occurs to me in the solution of this problem is the condition of the patient. I have no difficulty in treating the case; it is only diagnosing the condition of the patient that gives me pause. The discussion here, it seems to me, is chiefly regarding capping when the condition is normal. But what is to be done when the exposed pulp is diseased; the condition not healthy? Like Dr. Black, I attach great importance to the fact that the pulp diminishes as age advances.

*The Chair* (Dr. Noyes)—This is the most satisfactory discussion of the treatment of exposed pulps that I have ever heard. Diverse views are here expressed, and the methods of treatment here advocated are somewhat varying, but there is, I think, a general harmony on the main issue. In my view the conditions governing the treatment of the pulp are readily ascertainable; the same, in fact, as the other tissues of the body. There should be simply antiseptic treatment with the least possible irritation, and a careful covering up of the exposed parts. Oxy-chloride of zinc, for instance, stimulates, but whether it overstimulates is the main problem. These principles are the key to the matter, and the apparently diverse views here expressed are reconcilable, I think, if it be considered that good judgment and careful methods underlie all. (Applause.)

*Dr. Crouse*—I call upon the man who converted me to give his views. There he stands—Dr. Cushing.

*Dr. Cushing*—I have little to say. This has been a good discussion, but one or two points may, perhaps, be elaborated. I was pleased with the position taken in the opening of the paper—that the condition of the patient should govern and determine the question of capping or not capping. Doubtless, many pulps ought not to be capped. Dr. Newkirk has well emphasized the necessity for skillful use of good instruments. I have, myself, had considerable experience in the capping of pulps, and I believe that by judicious treatment they can nearly always be saved. My experience is that the method is successful even with children. As to age, it is doubtful if it should be performed when the patient is over thirty-five years of age; it is then liable to be unsuccessful; but at forty I think failure is almost inevitable. Also, where there has been inflammation of the parts since maturity, success in capping is very doubtful.

*Dr. Black*—We must not, I think, consider that success is doubtful because there are some failures. I spoke of cases where patients came with pulps already dead, though not exposed. There may be incipient or chronic abscess, but the process of capping is ordinarily successful.

*Dr. Newkirk*—Regarding the condition, I would say if the patient is healthy or has a smooth skin, that betokens a good condition. But I insist that the patient should be fully informed of the nature of the operation, and that the chances of failure are at least ten per cent. I think an error often obtains in removing the temporary filling too quickly after the pulp has been capped. It is better to leave it much longer; then there is less liability to after complaint.

*Dr. Spalding*—I do not like to have this interesting discussion closed without emphasizing one point, and that is, the importance of preserving the pulp in young subjects. Up to, say, sixteen years of age, the extirpation of the pulp and the filling of the root canals is a very uncertain operation. Young practitioners may infer that they have a license to destroy pulps at any age. The age of the patient is an important consideration in determining the treatment.

*Dr. A. W. Harlan*—Closed the discussion. He was of the opinion that pulps are very frequently capped too hastily. A patient calls, and the dentist having nothing else to do, perhaps, immediately caps the pulp; and this with the expectation of success. This is all wrong. A course of preliminary treatment is necessary. Nor did he believe in exposing pulps. He did, however, believe in trying to save them—especially the pulps of young persons. We should make the attempt in any case.

As an antiseptic, his experience led him to commend iodoform mixed with oil of cinnamon, by which the odor was rendered less pungent. So prepared, iodoform was not only a good antiseptic, but a stimulant as well. Regarding the treatment, he preferred oxyphosphate of zinc as the capping material. This should be protected and kept in position by a gutta percha filling, very carefully introduced. This prevents the washing away of the cap before hardening. At the close of his remarks the society adjourned.

WEDNESDAY MORNING SESSION.

Upon the calling of the regular order, Mrs. Dr. Kate C. Moody, of Mendota, read an entertaining essay on "Reflex Pain," which elicited much favorable comment. She was the only lady dentist present who participated in the proceedings. She considered pathological conditions productive of real or true pain, and compared the same with reflex or sympathetic pain. She regarded irritation as a chief cause of real pain, and stated that reflex pain was readily experienced in its effect, but its causes could not be easily determined. The relations of sympathetic nerves were frequently established through the existence of pain. Neuralgia, in its various forms, was instanced as productive of a large degree of real pain, and to disordered blood could doubtless be attributed much of the nervous disturbance which might be classed under the head of reflex pain. The essayist urged the cultivation of knowledge of the real causes of pain, to the end that the distress thus engendered might be speedily and effectively alleviated.

*Dr. A. W. Freeman*—Had frequently noticed reflex action arising from the dental organs afflicting the pneumogastric nerve; said inflammation was always perplexing when the cause was not apparent. He was also greatly surprised at the workings, at times, of certain remedies. He would cite a case that caused him a great deal of perplexity. The case was that of an apparently sound person, with apparently sound teeth, suffering great pain. On examination he found the teeth were really so loose that with his thumb and finger he could pull them out. Of sympathetic cases this one was the most remarkable he had ever seen. He asked the lady to call again. The pain was traceable to the reproductive organs, influenced especially by first conception. In a couple of months the teeth became firm.

He had a second case, that of difficult dentition. There was diarrhœa present, with great difficulty in checking the bowels, as there was much irritation of the lower passage. Finally he prescribed laudanum mixed with castor oil. There was chorea all this time. Pressing hard against the gum he cut it, and soon the tooth, a canine, made its appearance, and the child recovered. There is often observed in cases brought for treatment irritation of the dental organs, and a sort of muscular twitching simulating chorea. He had noticed it in a little grandson of his own, when suffering pain. He himself had a habit of muscular twitching at times.

*Dr. Black*—Said he would note one point, and try to be brief. He referred to the close kinship or relation between reflex pain and reflex motion, or motor disturbances, especially of the vaso-motor system. It is generally conceded that neuralgia is the result of deficient blood supply, yet it is singular that we may have neuralgia in persons not anemic. These points do not coincide. He knew very well that we understand reflex motion better than we comprehend reflex pain. He supposes these reflex pains, which are of the order of neuralgias, are brought about through the lack of blood caused by vaso-motor disturbances. The probability is that the pain is due to the condition of the vessels that supply the blood. Local tension may produce local neuralgia. He thinks, therefore, that it may be stated as a principle that neuralgia is the result of local tension.

The doctor cited the case of a patient unable to maintain an upright position. Careful examination disclosed that the tension was an insufficiency of blood on one side, so an upright position was untenable. The same remedy applicable in neuralgia will apply here; in fact, will apply in almost all cases not neuralgic, as well as in neuralgia produced by over tension. Absolutely we know nothing about these things. We can only construct our hypothesis to agree with the facts.

*Dr. Townsend*—Do you hold that, the cause of pain being on one side of the jaw, it will affect the other?

*Dr. Black*—Yes.

*Dr. Patrick*—We see by the latitude this discussion has taken how difficult it is to confine or circumscribe a specialty. "No pent-up Utica contracts our powers."

The movements which are caused in the nerves of special sense by external agents are numerous; in fact, it is external influence only that excites the organs of special sense to action. It is an ultimate fact in nature that consciousness is the result of stimulated nervous tissue; we know that we smell with our noses and see with our eyes; we cannot smell with our ears or see with our tongues; and yet, the gustatory and auditory nerves are in substance like the optic and the olfactory. The difference in function is evidently in the arrangement of the nerve cells and the disposition of the channels through which external agents have to travel to reach them. We constantly feel in spite of ourselves, for it is impossible for us to avoid having sensation when any object excites a nerve; the sensation is within us, but is dependent on external influences; we receive it, but how? It is evident that there is no connection between the words which I utter and the impression which my words have upon your brain; and how diversely the same words will impress different individuals! Witness the effect produced on the mind and body of an individual surrounded by a gay and happy company, who receives a telegram from the hand of a messenger, the few lines of which convey to him the intelligence that a great calamity has befallen his nearest and dearest friend. The shock throws him into convulsions; his friends endeavor to pacify him, but he is completely prostrated; a fever sets in and he is delirious for weeks, but finally recovers; his friends sympathized, but were not afflicted in the same manner; they saw the same characters on the scrap of paper that were seen by their unfortunate friend, and the intelligence was conveyed to their minds in precisely the same way. Yet how different the result on the nervous system.

Again, two individuals are engaged in conversation. Presently one utters words that jar upon the auditory nerves of the one spoken to; the vibrations pass from the nerves of special sense and seize upon the motor system of nerves; these contract the fibers of the levator muscles and suddenly pass the impulse to the extensors, which extending the arm with great force knocks the speaker down; and what for? simply for agitating a little air, the waves of which, passing to the auditory nerves of the one spoken to, were reflected back to the speaker in the form of a repulsive blow. Why should not this phenomenon be classed as an instance of reflex pain? The property of a nerve cord to receive an impression and conduct it inwards is common to all nerves; but there are certain nerves that not only have the property of receiving and conducting an impulse inwards, but also have the power to return the same impulse outwards; these are ganglionic nerves, and are distinguished from all others by nervous knots, composed of gray matter, which knots distinguish them, physiologically, as central organs, and endow them with the power of reflex action, whether of pain or pleasure. The reflex power is possessed by the gray matter only, and not by the white substance of the cord. The afferent impulse being converted into an efferent impulse, producing what is called reflex action, is beautifully illustrated in the expansion and contraction of the iris in accommodating itself to the luminous vibrations which produce the sensation of light, when the light passes through the central opening or pupil, and thence through the lens to the retina, where it is received by the nerves of the retina on its concave surface; now, if the light be too strong, the small ganglia connected with the nerves which control the movements of the iris, instantly transmit an efferent impulse, which contracts the pupil and shuts off the light; if, however, the luminous vibrations are not sufficient to produce a strong sensation of light (as in a dark room), the iris expands and the pupil is enlarged by the same process of reflex action. The points of a star-fish are supplied with nerves and ganglia to produce a reflex action; touch one of the extended points and the impulse is carried toward the center to the ganglia controlling the point touched; at once the ganglia return the impulse to the muscles, which immediately contract, and the point curls up. A similar phenomenon is produced when a frond of the sensitive plant is touched; the frond, however, having no muscles to contract, simply droops. Are those ganglia that we find in plant and animal, that bring about such positive action, endowed with intelligence? or are all these phenomena merely a manifestation of chemical and mechanical energy? When we pass beyond the boundary of physics into the region of metaphysics, we become as men born blind discussing the nature of light.

The speaker exhibited a tooth with an exostosed root, showing an elongation of the root rather than an enlargement of its diameter, adding that this process had gone on, although the connection of the nervous system with the pulp had long been severed, as was proved by an examination after extraction.

*Dr. Conrad*—Inquired if visitors were privileged to speak, and being assured in the affirmative, said that, in his view, in discussing reflex pain there was a confusion of terms, and it should be reflex action; not a pathological condition as described by the essayist, but rather physiological.

On motion the subject was passed.

(TO BE CONTINUED.)

